# Supplementary figures and images for: Heat shock factor regulation of antimicrobial peptides expression suggests a conserved defense mechanism induced by febrile temperature in arthropods
Source: eLife. 2026 Apr 16;13:RP101460. doi: 10.7554/eLife.101460 (PMC13086497; doi:10.7554/eLife.101460)

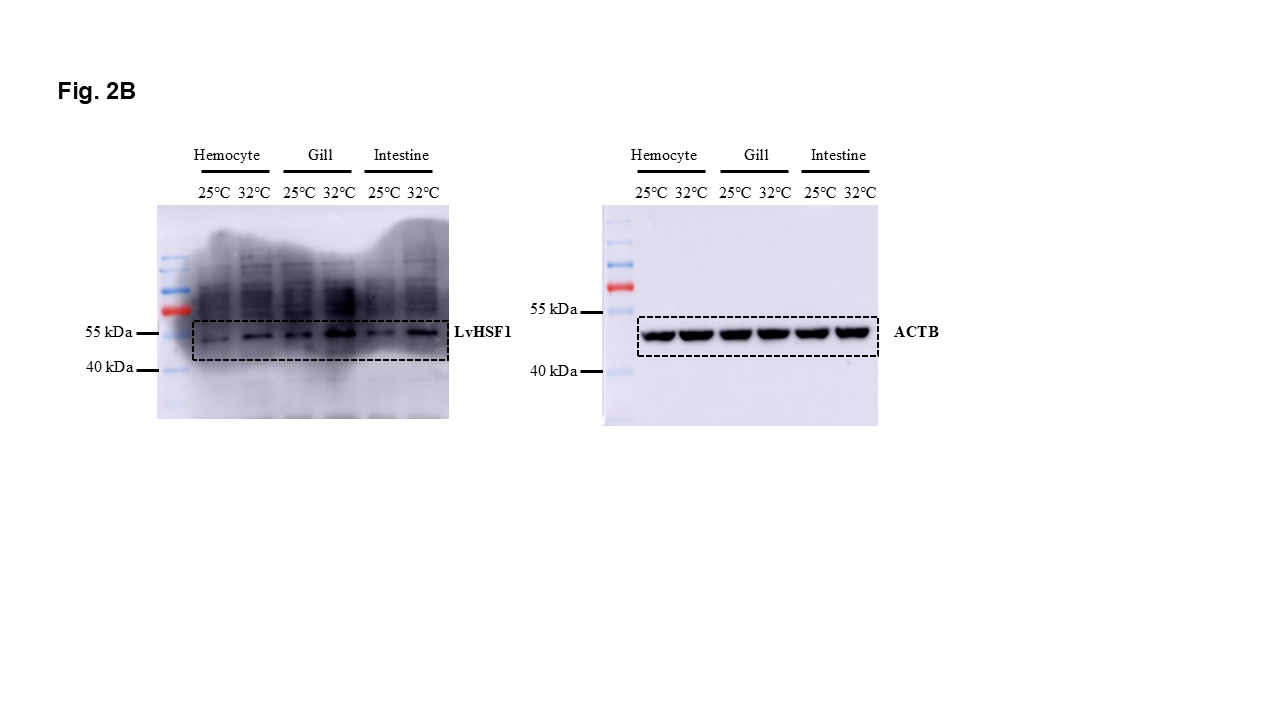

Supplement: Figure 2—source data 2. [file elife-101460-fig2-data2.zip › Fig. 2-source data2/Fig. 2-source data 2.tif]

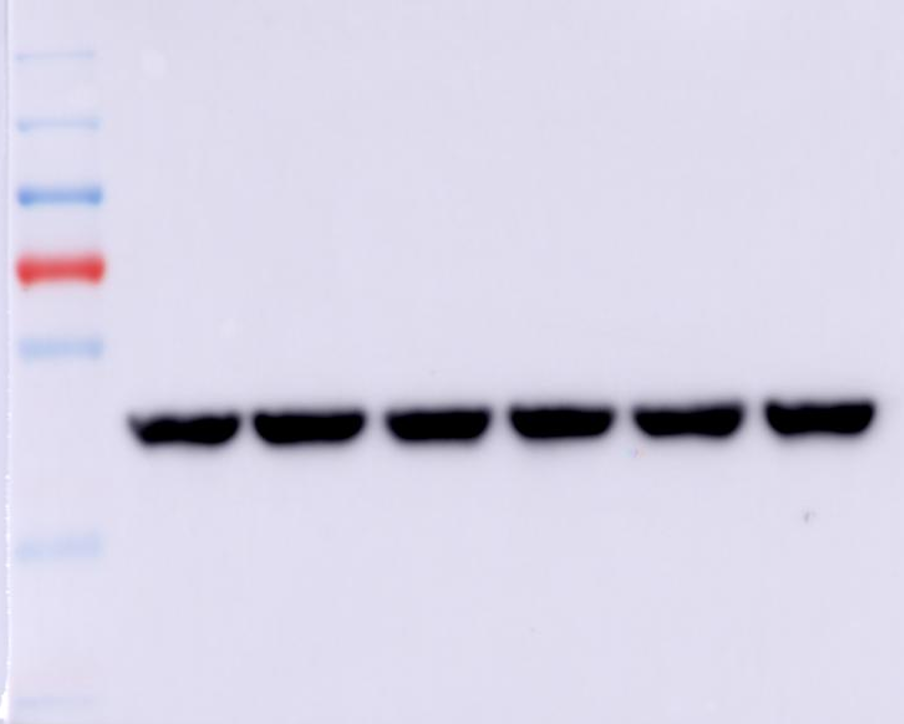

Supplement: Figure 2—source data 3. [file elife-101460-fig2-data3.zip › Fig. 2-source data3/Fig. 2B ACTB.tif]

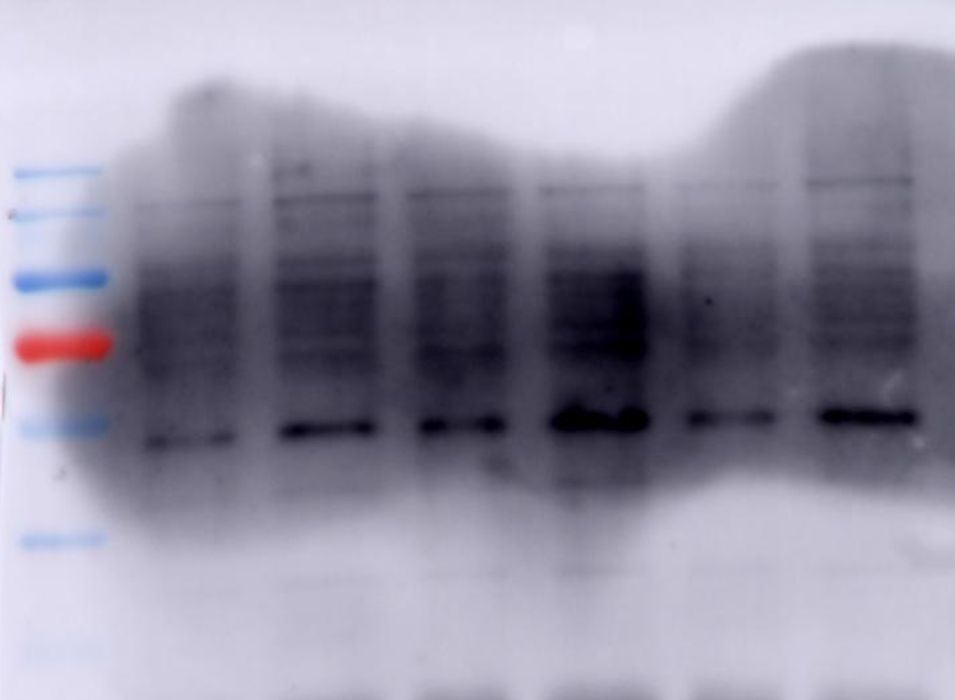

Supplement: Figure 2—source data 3. [file elife-101460-fig2-data3.zip › Fig. 2-source data3/Fig. 2B HSF1.tif]

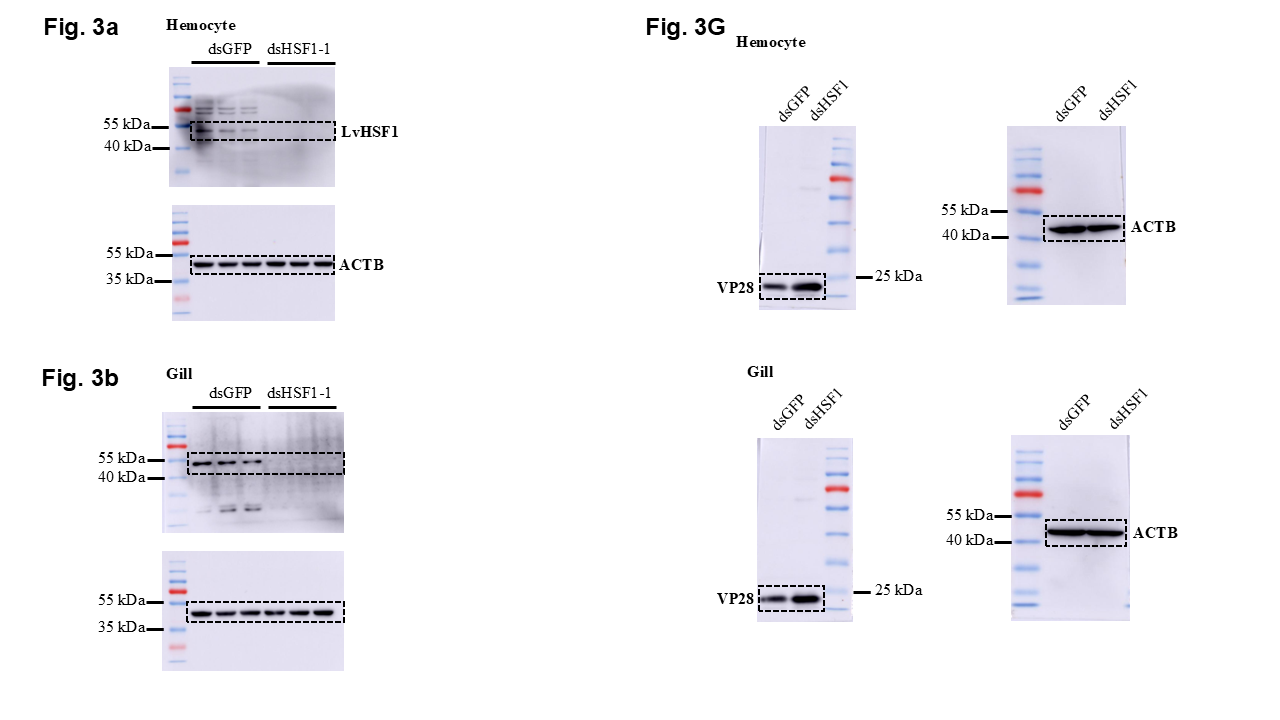

Supplement: Figure 3—source data 2. [file elife-101460-fig3-data2.zip › Fig. 3-source data2/Fig. 3-source data2.tif]

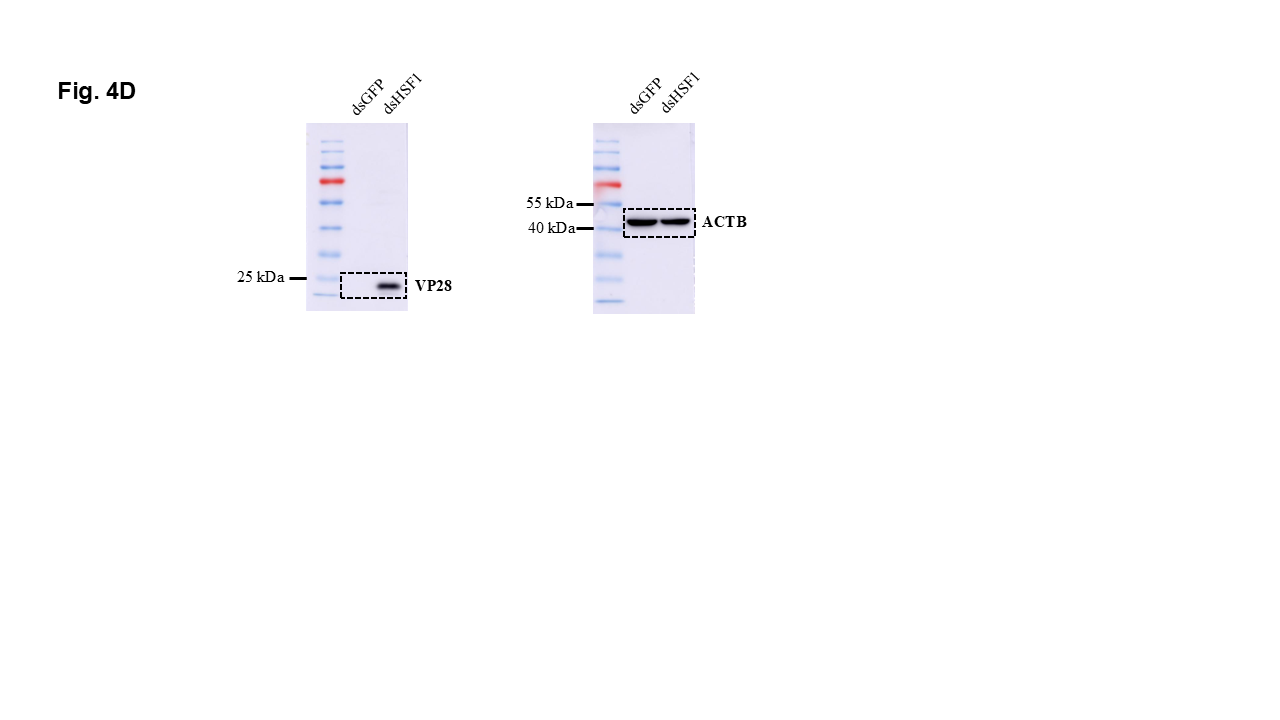

Supplement: Figure 4—source data 2. [file elife-101460-fig4-data2.zip › Fig. 4-source data2/Fig. 4-source data2.tif]

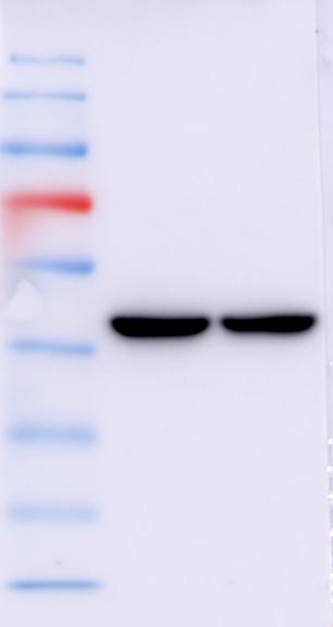

Supplement: Figure 4—source data 3. [file elife-101460-fig4-data3.zip › Fig. 4-source data3/Fig. 4D ACTB.tif]

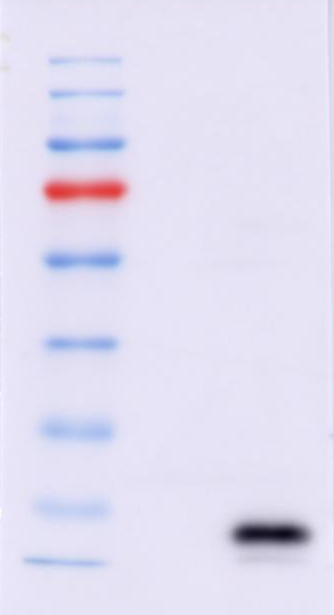

Supplement: Figure 4—source data 3. [file elife-101460-fig4-data3.zip › Fig. 4-source data3/Fig. 4D VP28.tif]

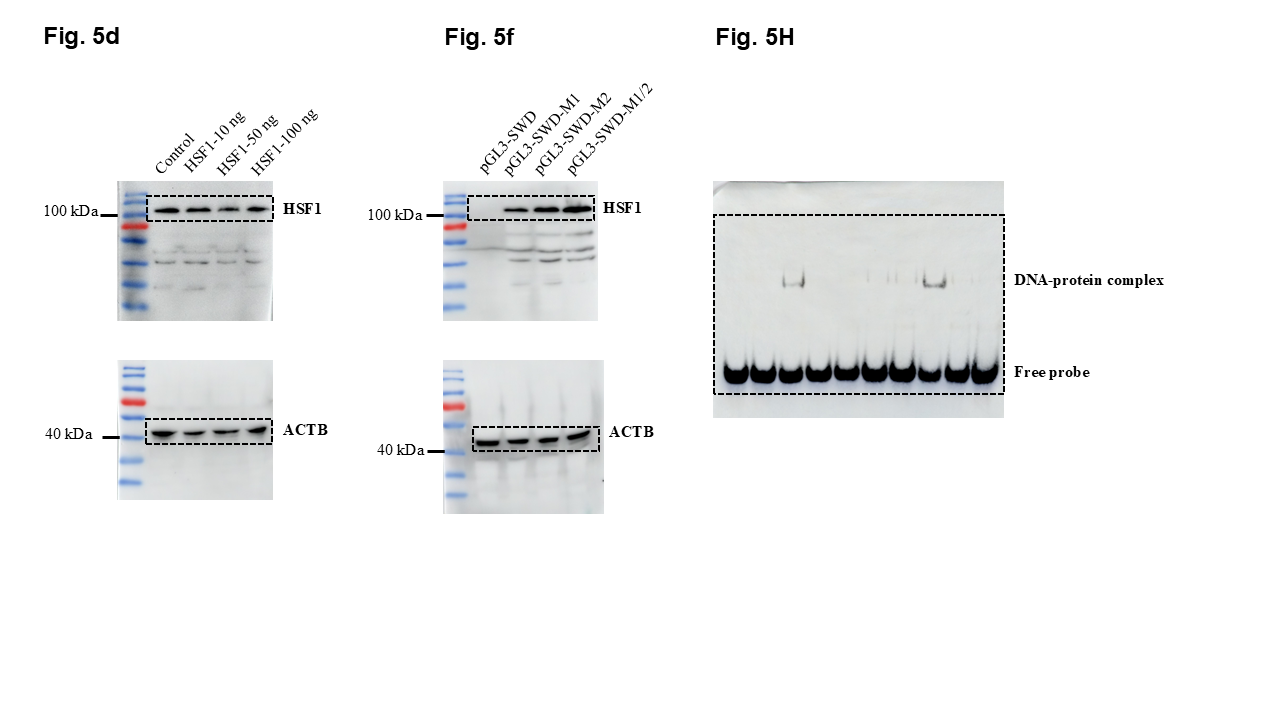

Supplement: Figure 5—source data 2. [file elife-101460-fig5-data2.zip › Fig. 5-source data2/Fig. 5-source data2.tif]

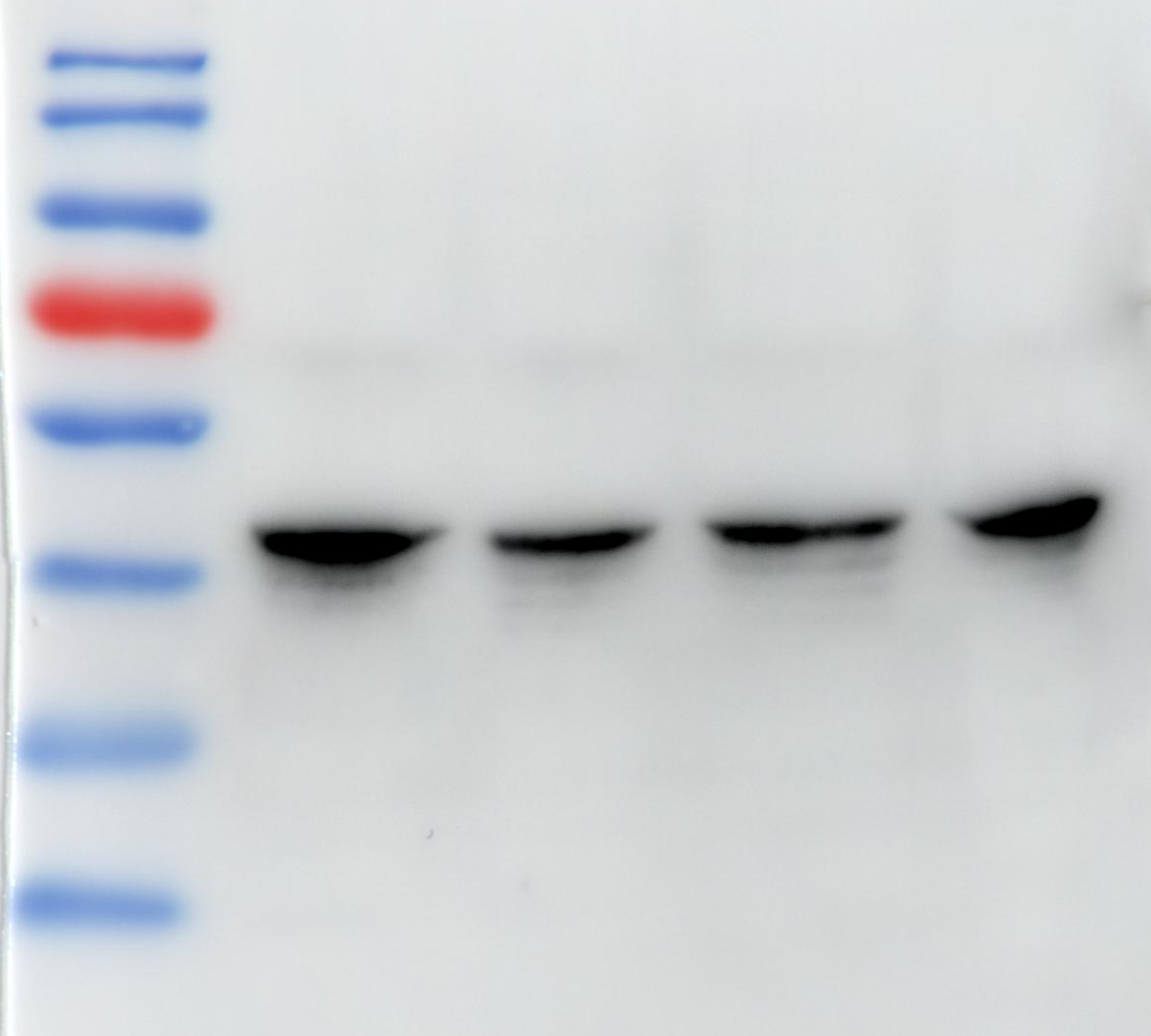

Supplement: Figure 5—source data 3. [file elife-101460-fig5-data3.zip › Fig. 5-source data3/Fig. 5d ACTB.tif]

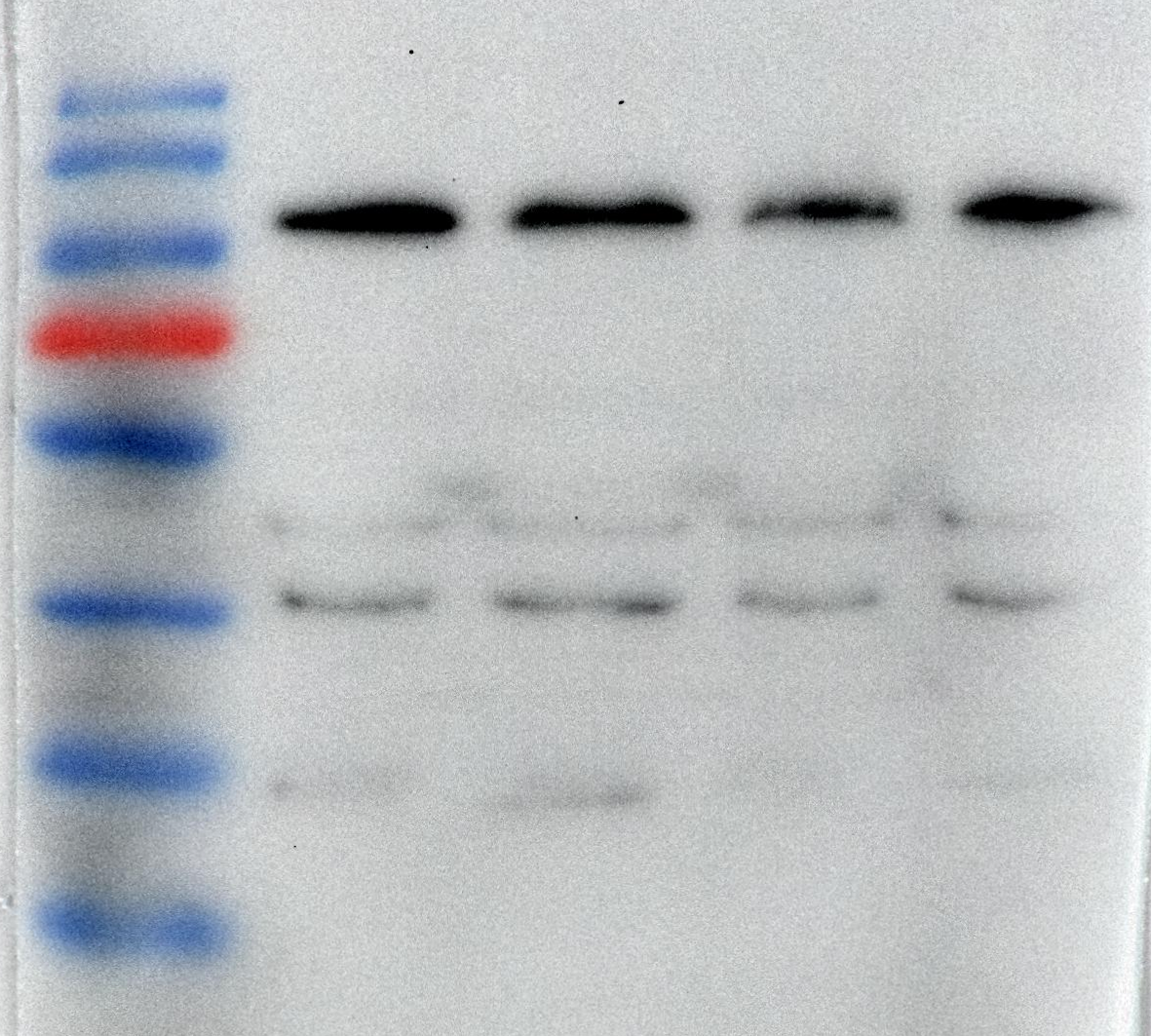

Supplement: Figure 5—source data 3. [file elife-101460-fig5-data3.zip › Fig. 5-source data3/Fig. 5d HSF1.tif]

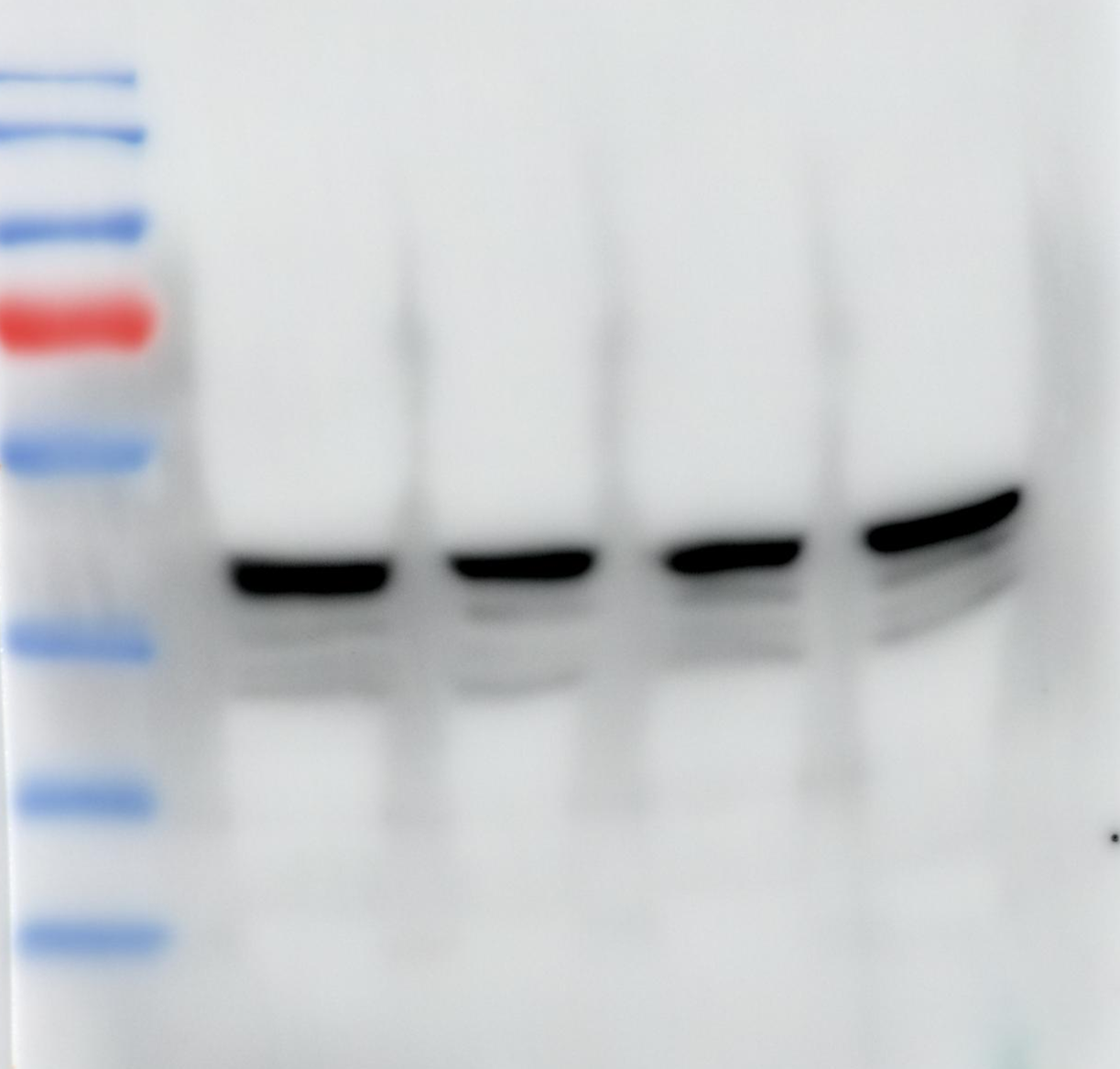

Supplement: Figure 5—source data 3. [file elife-101460-fig5-data3.zip › Fig. 5-source data3/Fig. 5f ACTB.tif]

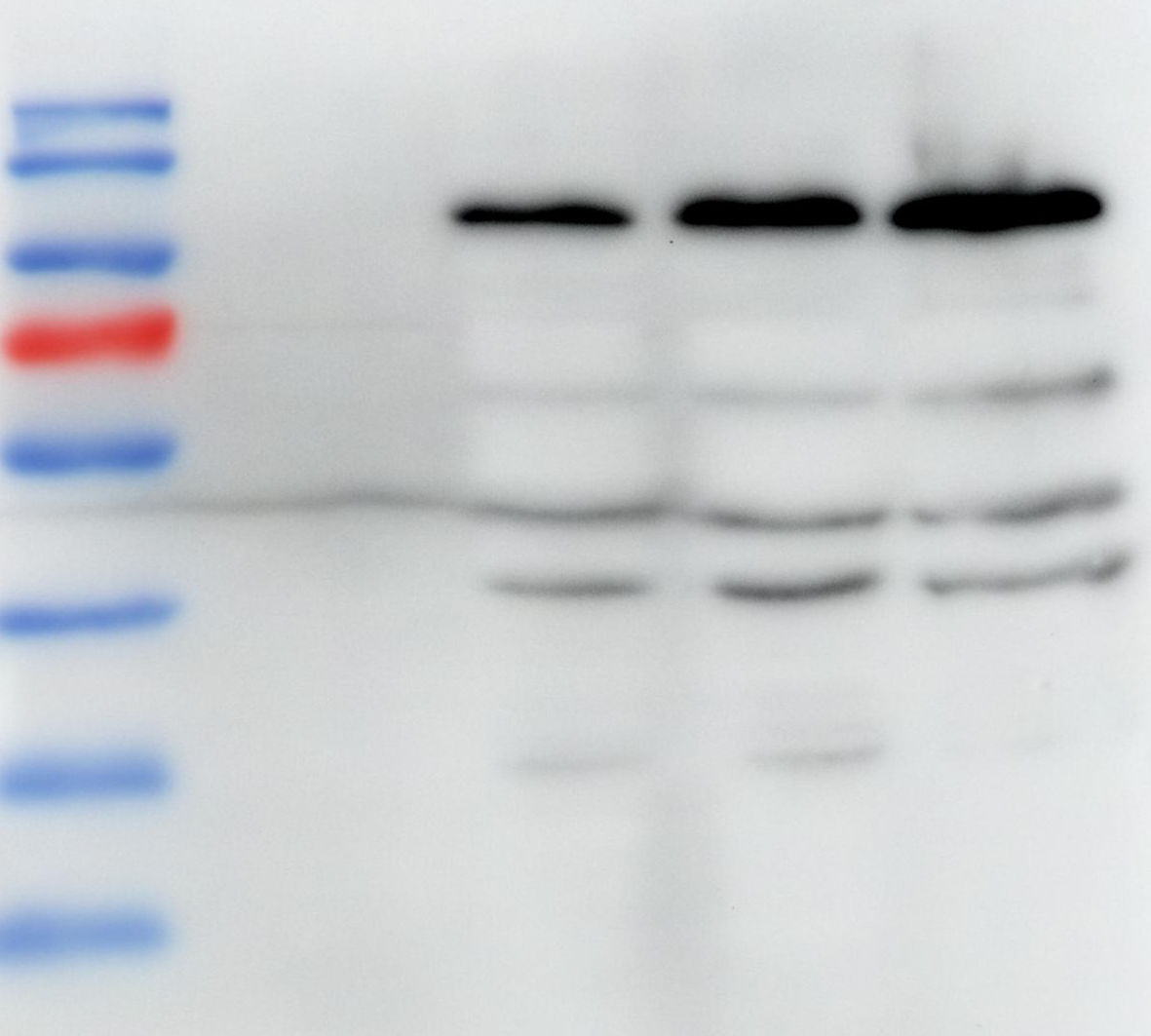

Supplement: Figure 5—source data 3. [file elife-101460-fig5-data3.zip › Fig. 5-source data3/Fig. 5f HSF1.tif]

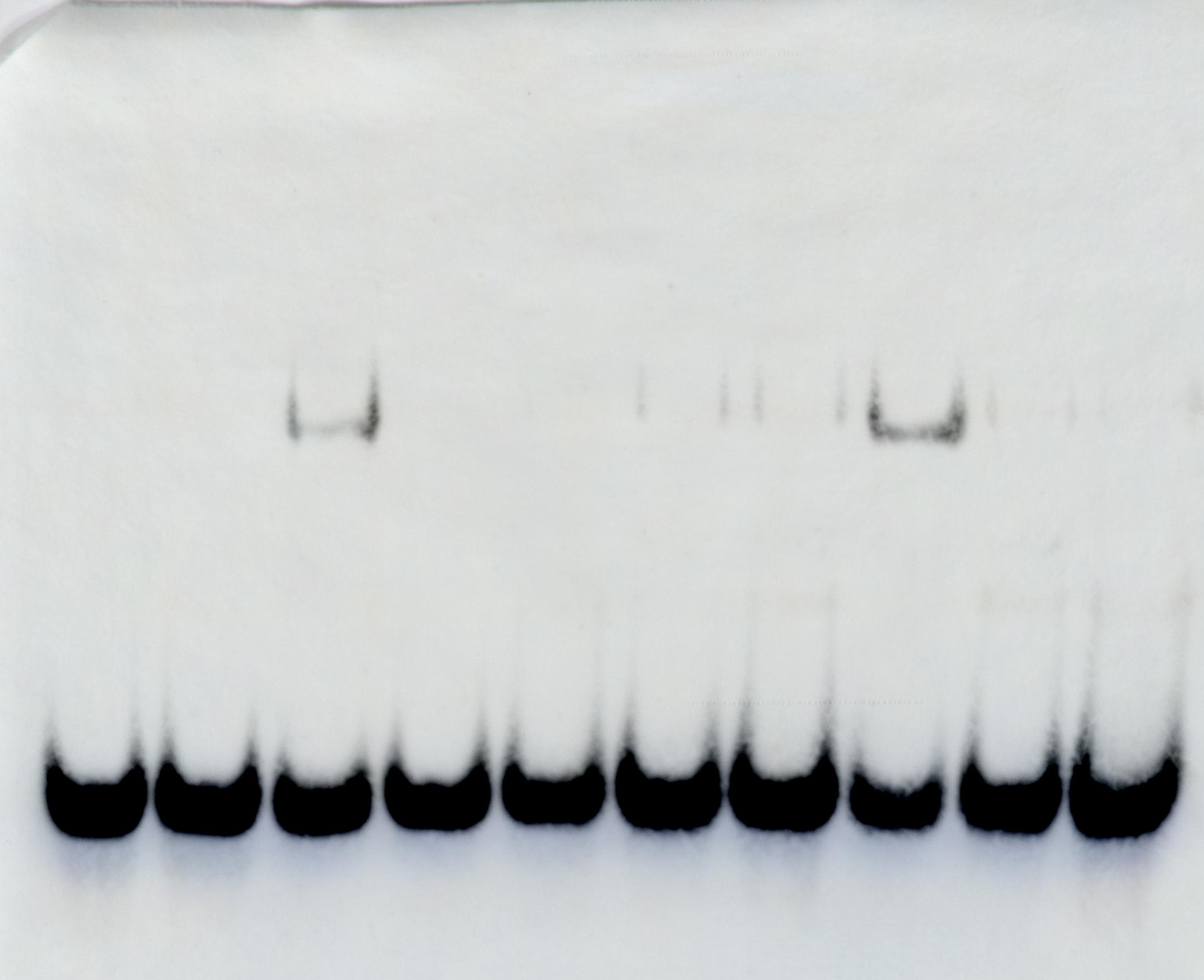

Supplement: Figure 5—source data 3. [file elife-101460-fig5-data3.zip › Fig. 5-source data3/Fig. 5H EMSA.tif]

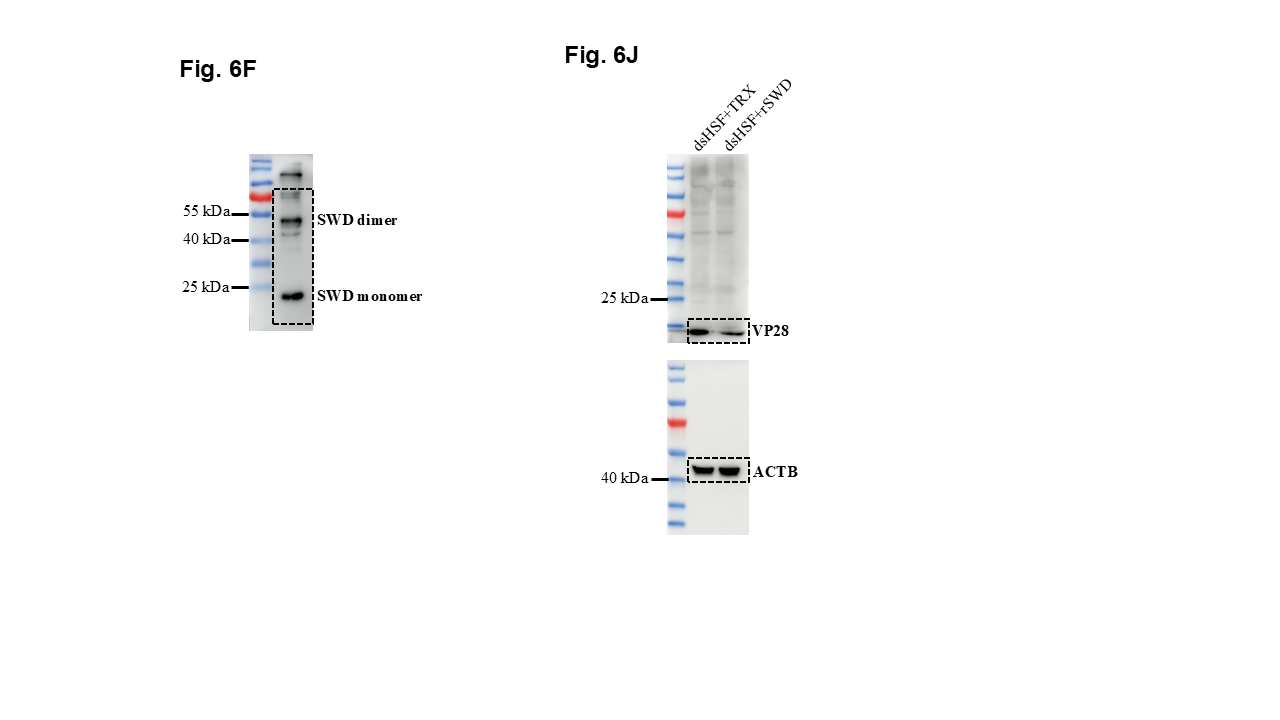

Supplement: Figure 6—source data 2. [file elife-101460-fig6-data2.zip › Fig. 6-source data2/Fig. 6-source data2.tif]

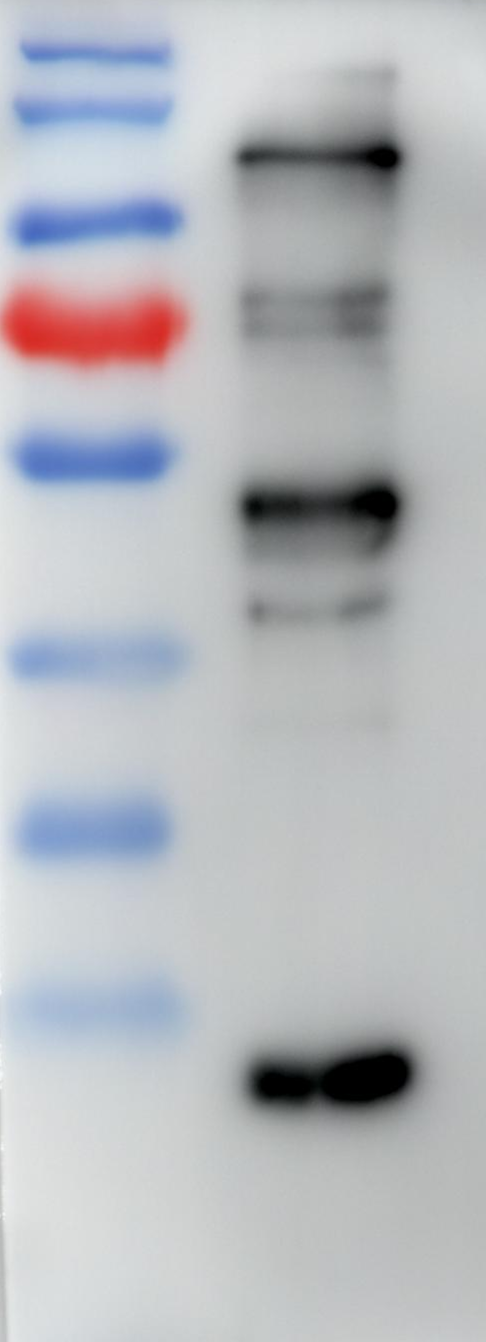

Supplement: Figure 6—source data 3. [file elife-101460-fig6-data3.zip › Fig. 6-source data3/Fig. 6F SWD.tif]

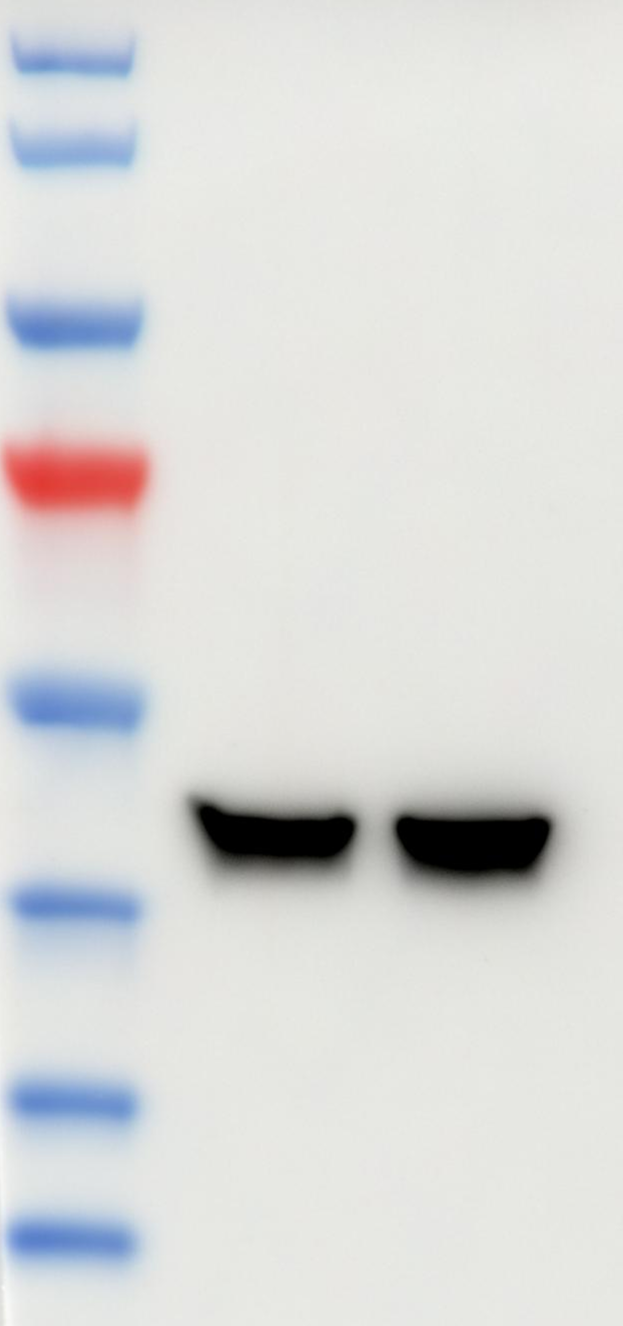

Supplement: Figure 6—source data 3. [file elife-101460-fig6-data3.zip › Fig. 6-source data3/Fig. 6J ACTB.tif]

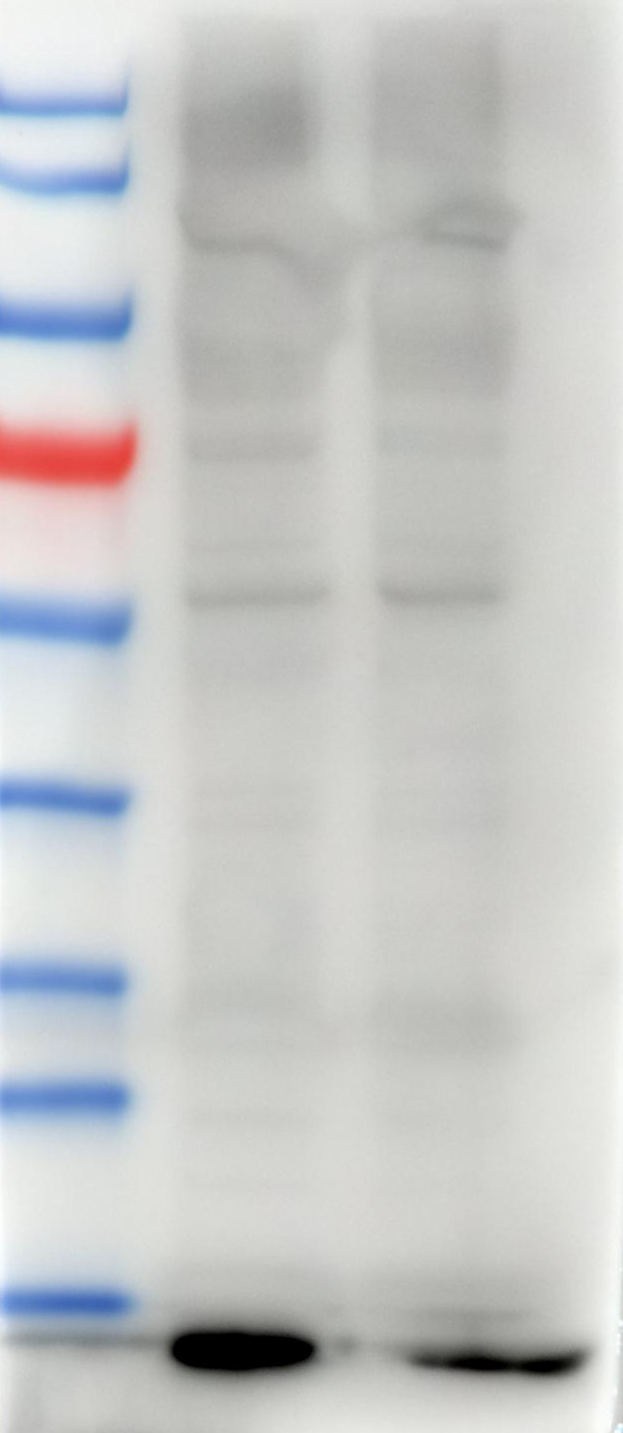

Supplement: Figure 6—source data 3. [file elife-101460-fig6-data3.zip › Fig. 6-source data3/Fig. 6J VP28.tif]

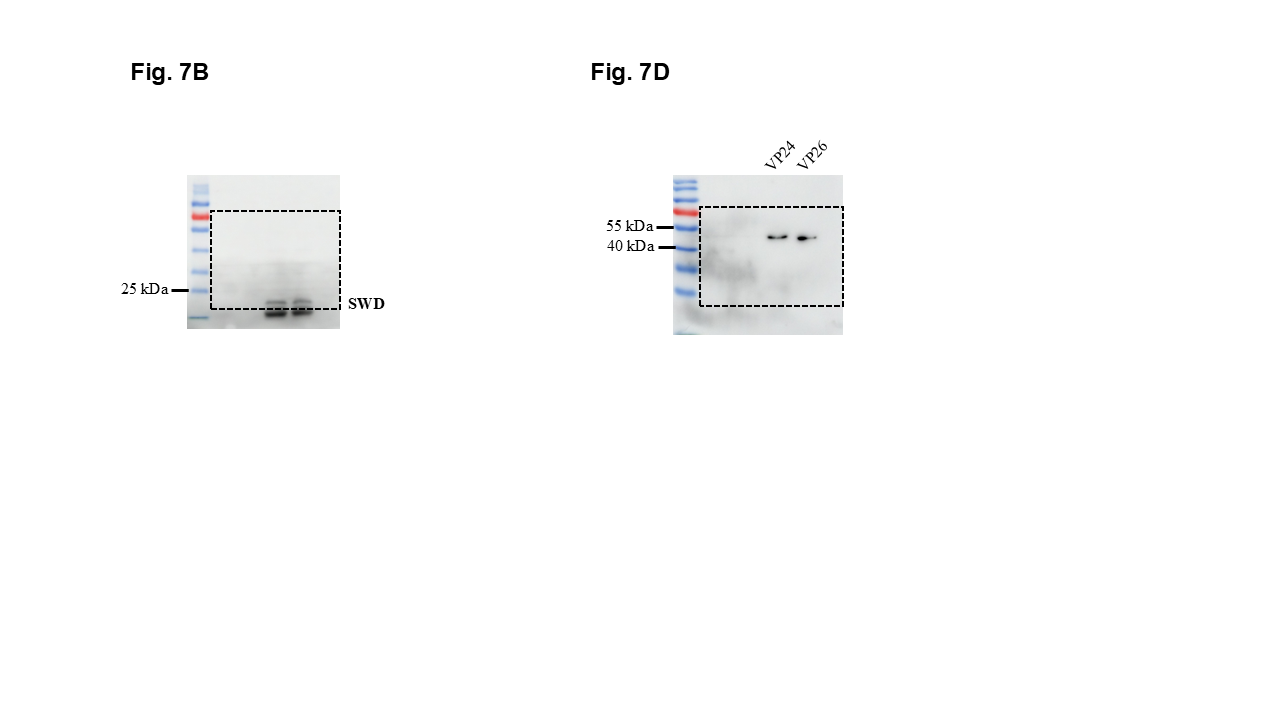

Supplement: Figure 7—source data 2. [file elife-101460-fig7-data2.zip › Fig. 7-source data2/Fig. 7-source data2.tif]

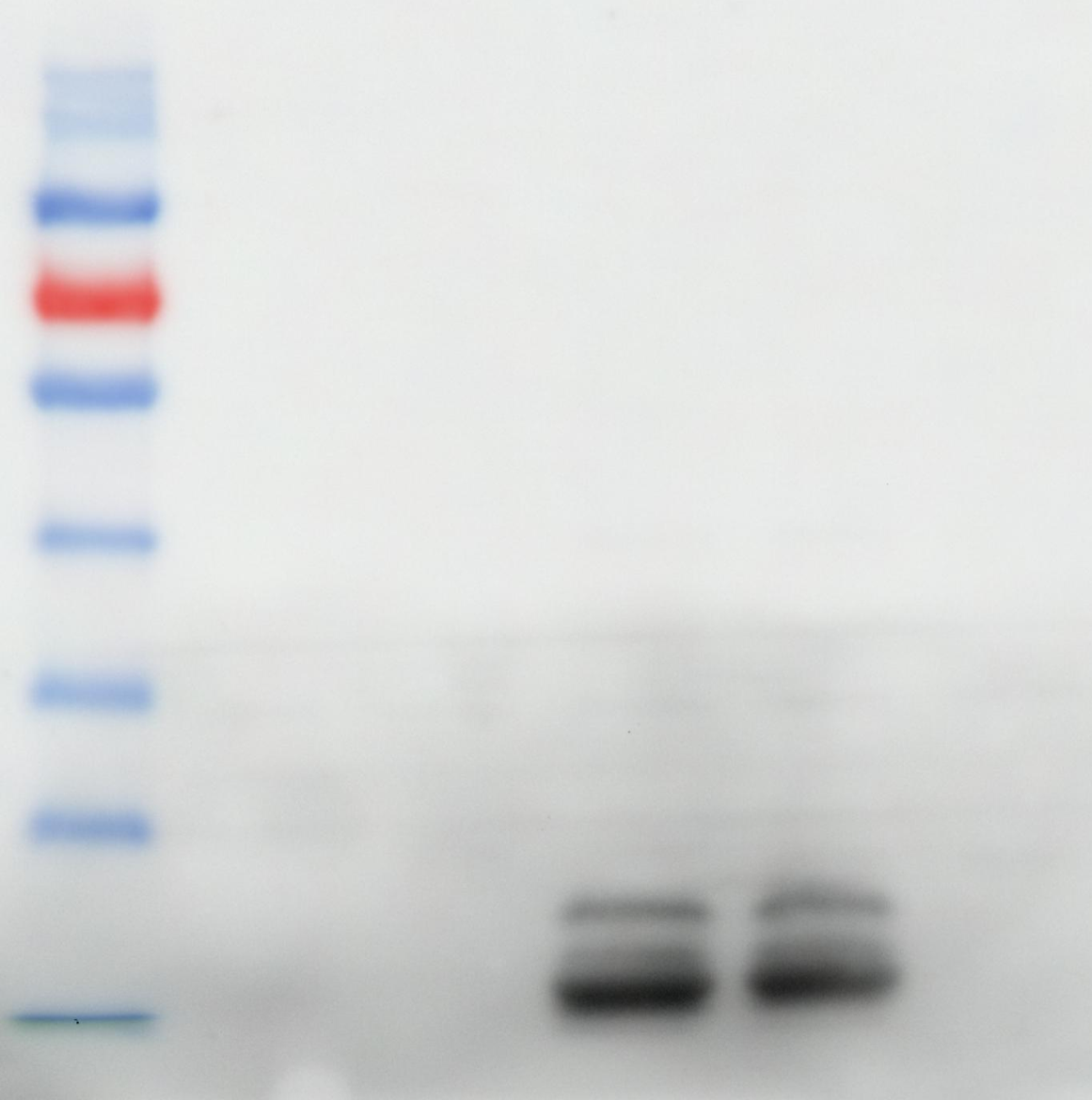

Supplement: Figure 7—source data 3. [file elife-101460-fig7-data3.zip › Fig. 7-source data3/Fig. 7B anti-6×His.tif]

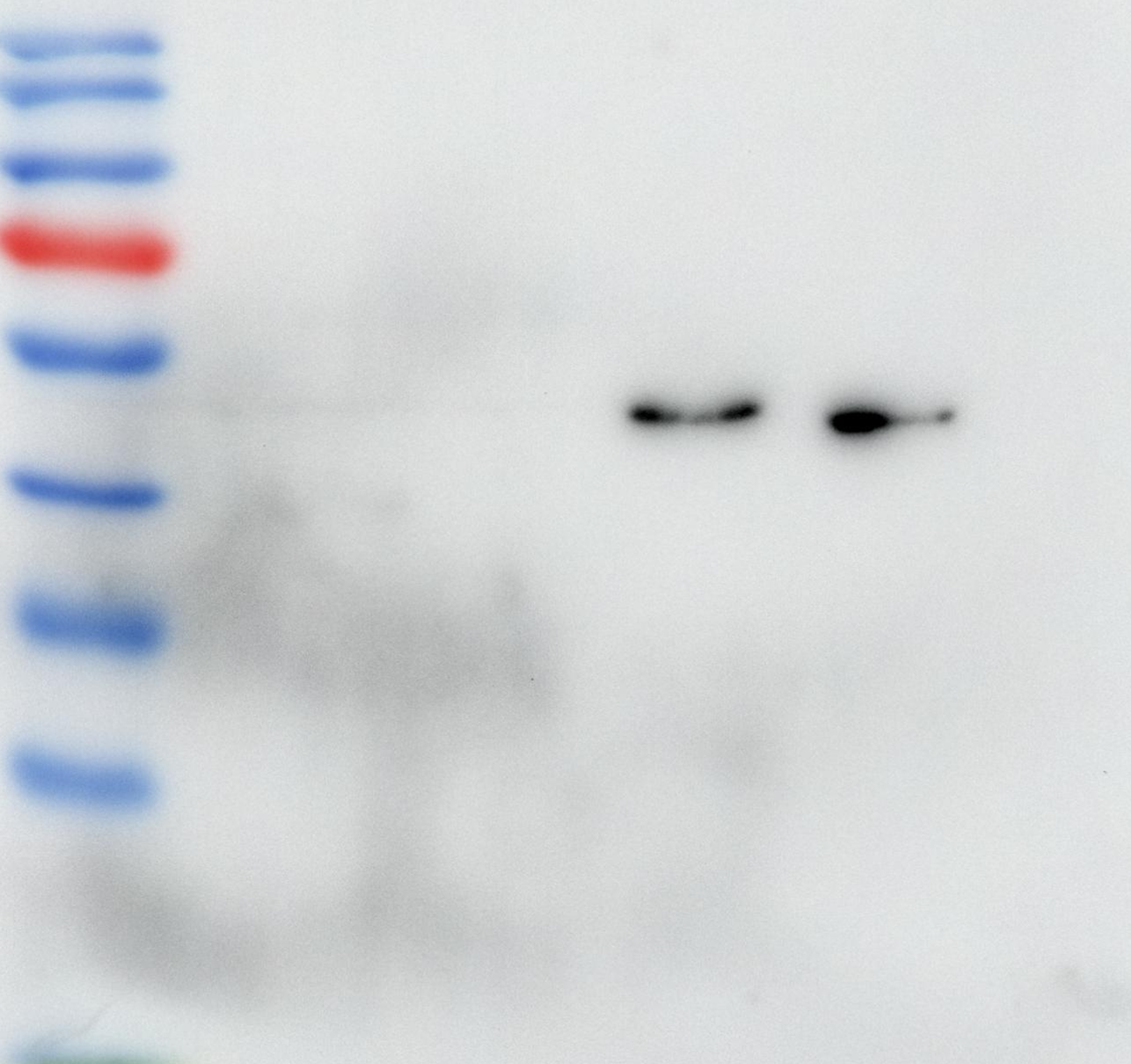

Supplement: Figure 7—source data 3. [file elife-101460-fig7-data3.zip › Fig. 7-source data3/Fig. 7D anti-GST.tif]

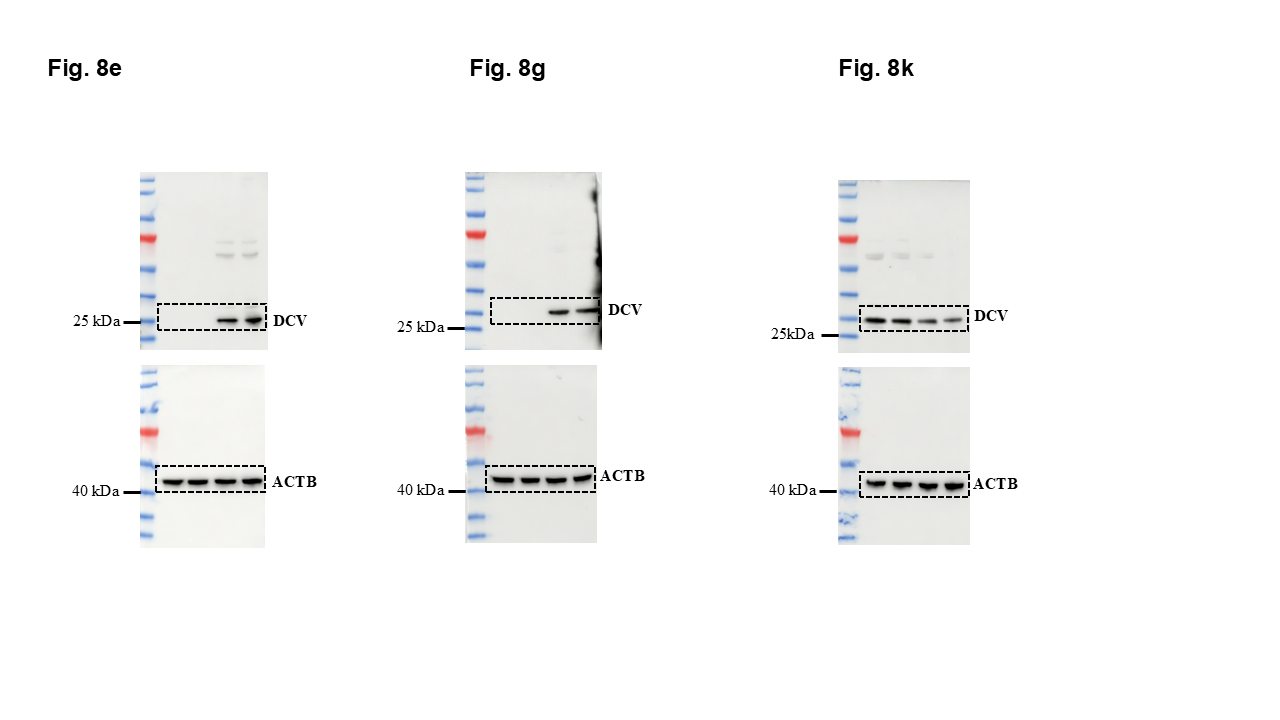

Supplement: Figure 8—source data 2. [file elife-101460-fig8-data2.zip › Fig. 8-source data2/Fig. 8-source data2.tif]

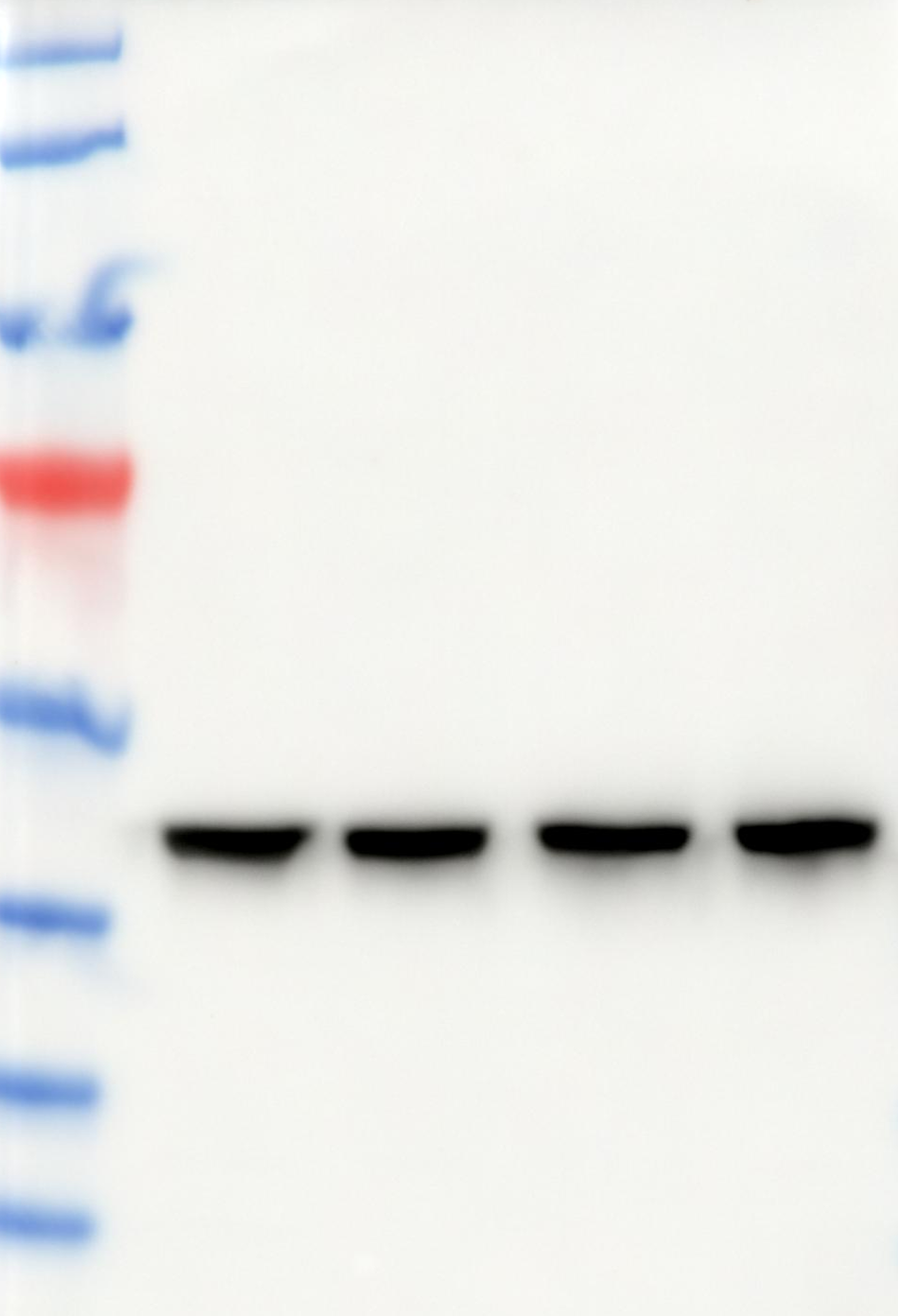

Supplement: Figure 8—source data 3. [file elife-101460-fig8-data3.zip › Fig. 8-source data3/Fig. 8e ACTB.tif]

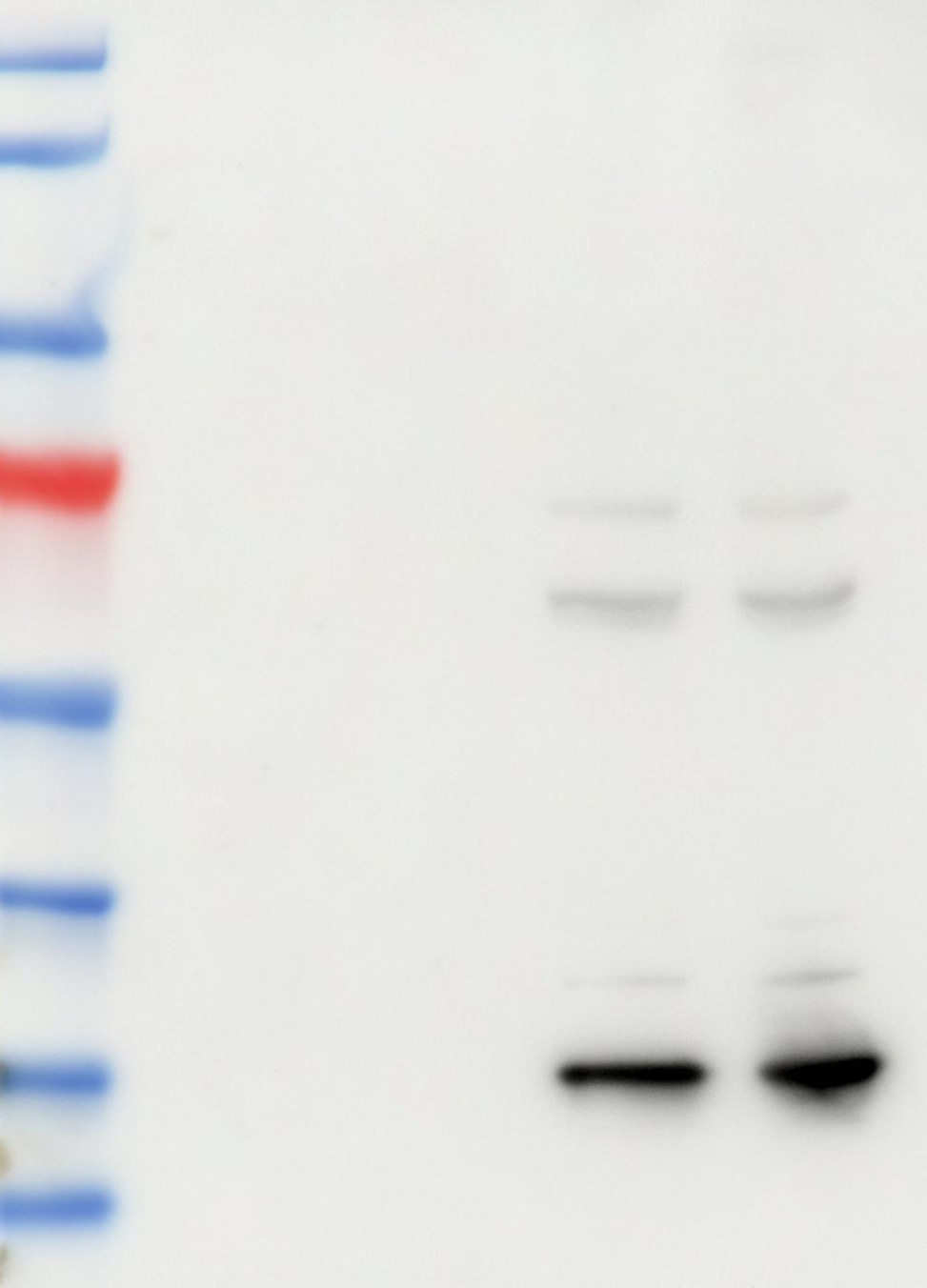

Supplement: Figure 8—source data 3. [file elife-101460-fig8-data3.zip › Fig. 8-source data3/Fig. 8e DCV.tif]

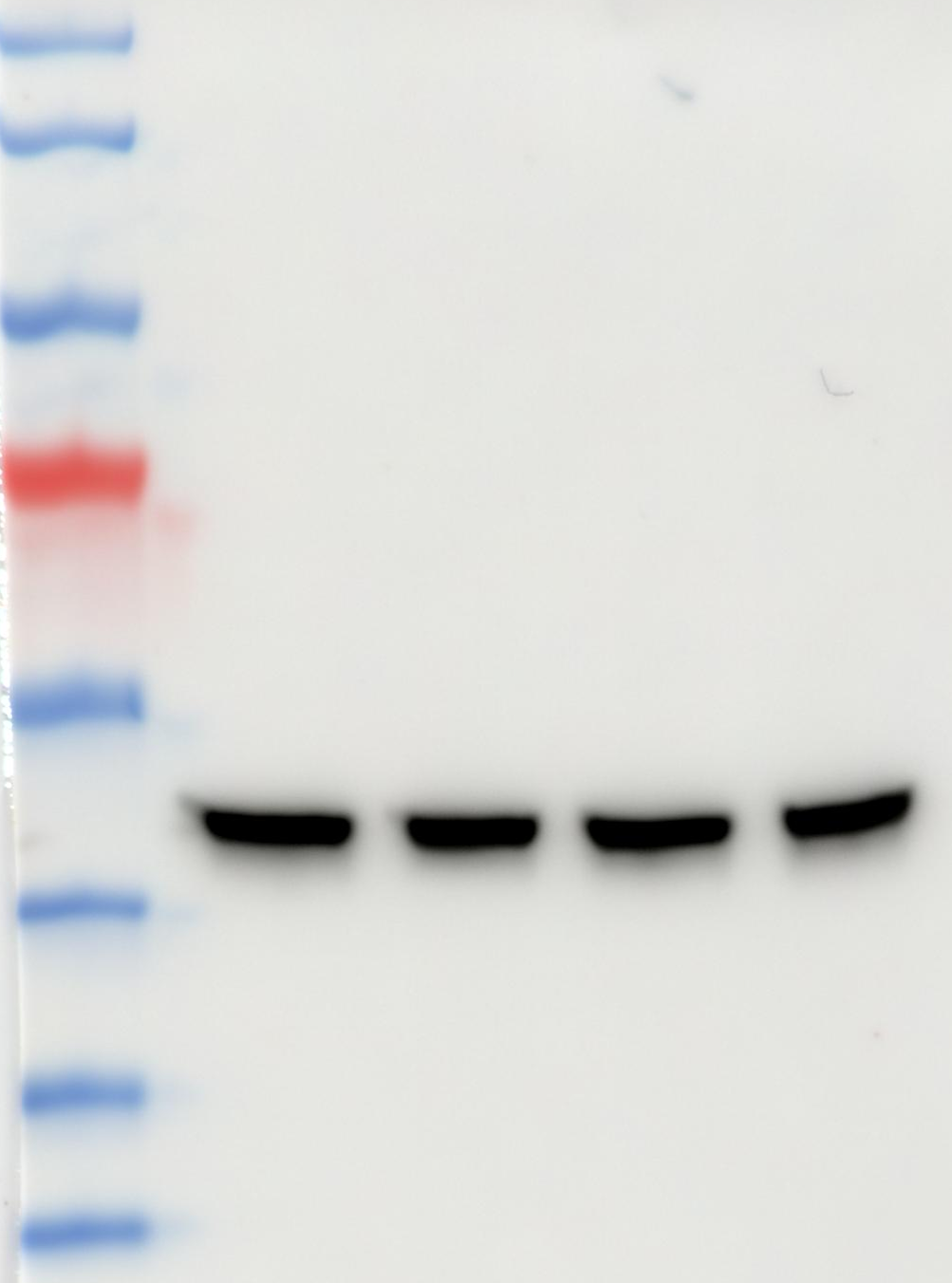

Supplement: Figure 8—source data 3. [file elife-101460-fig8-data3.zip › Fig. 8-source data3/Fig. 8g ACTB.tif]

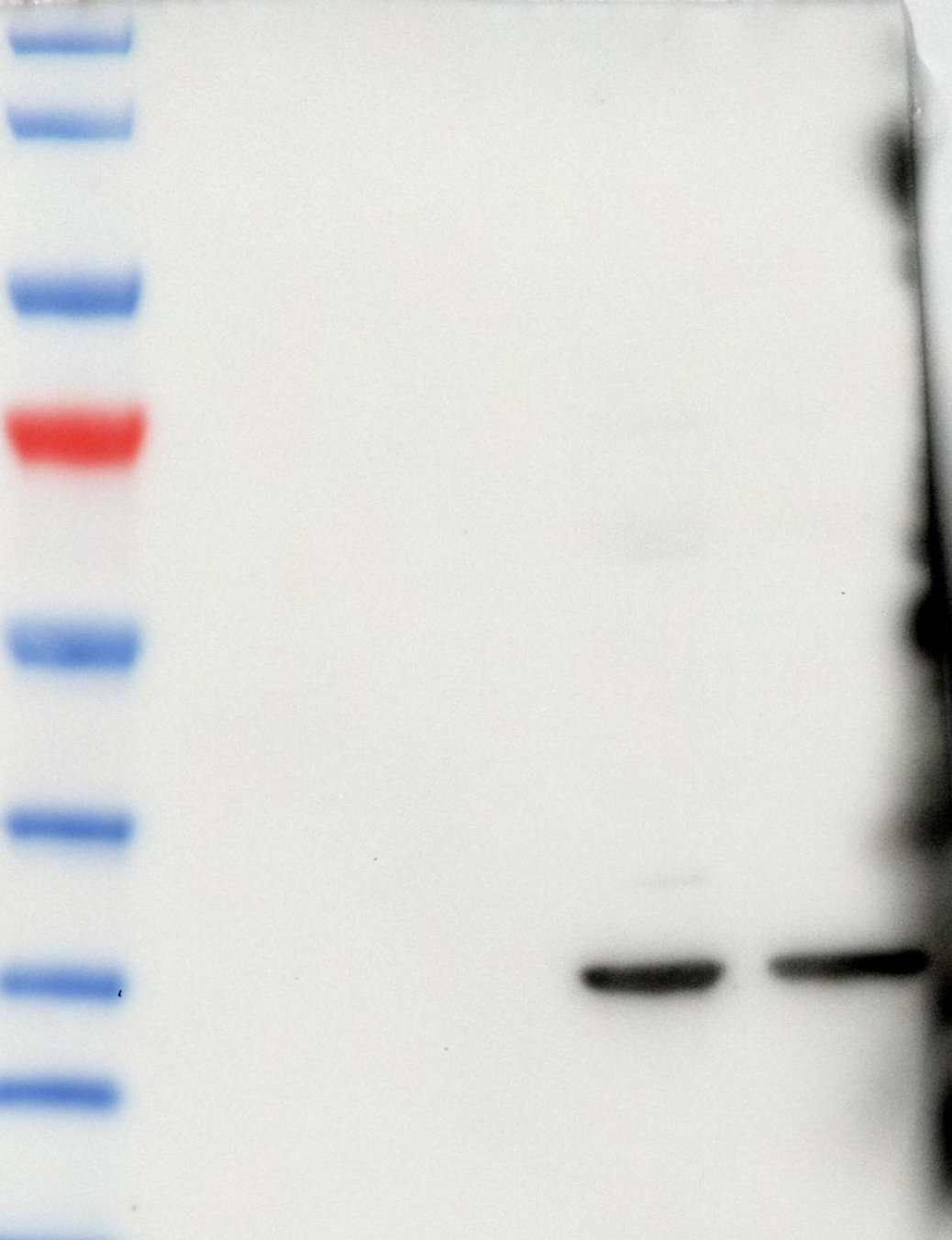

Supplement: Figure 8—source data 3. [file elife-101460-fig8-data3.zip › Fig. 8-source data3/Fig. 8g DCV.tif]

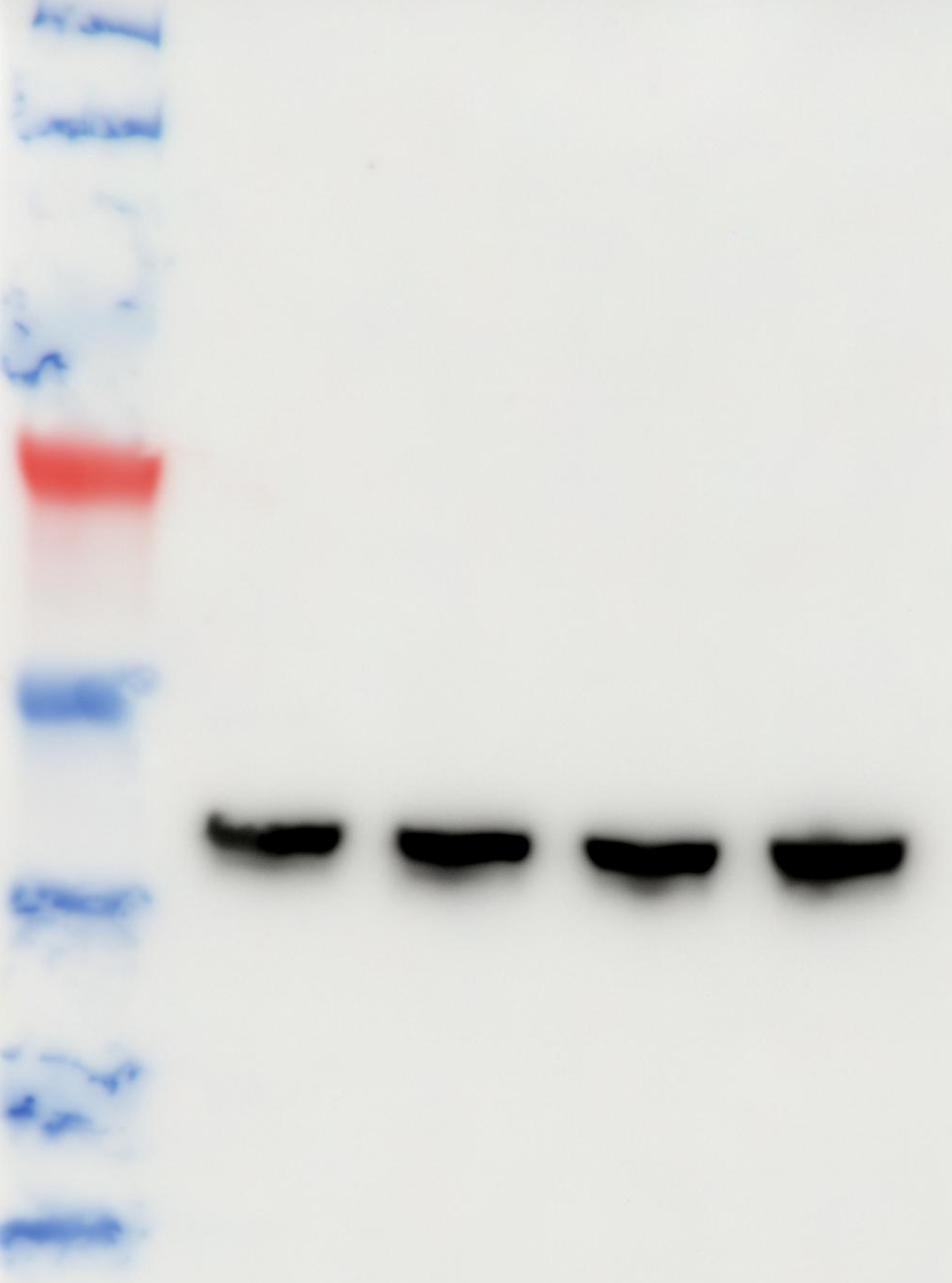

Supplement: Figure 8—source data 3. [file elife-101460-fig8-data3.zip › Fig. 8-source data3/Fig. 8k ACTB.tif]

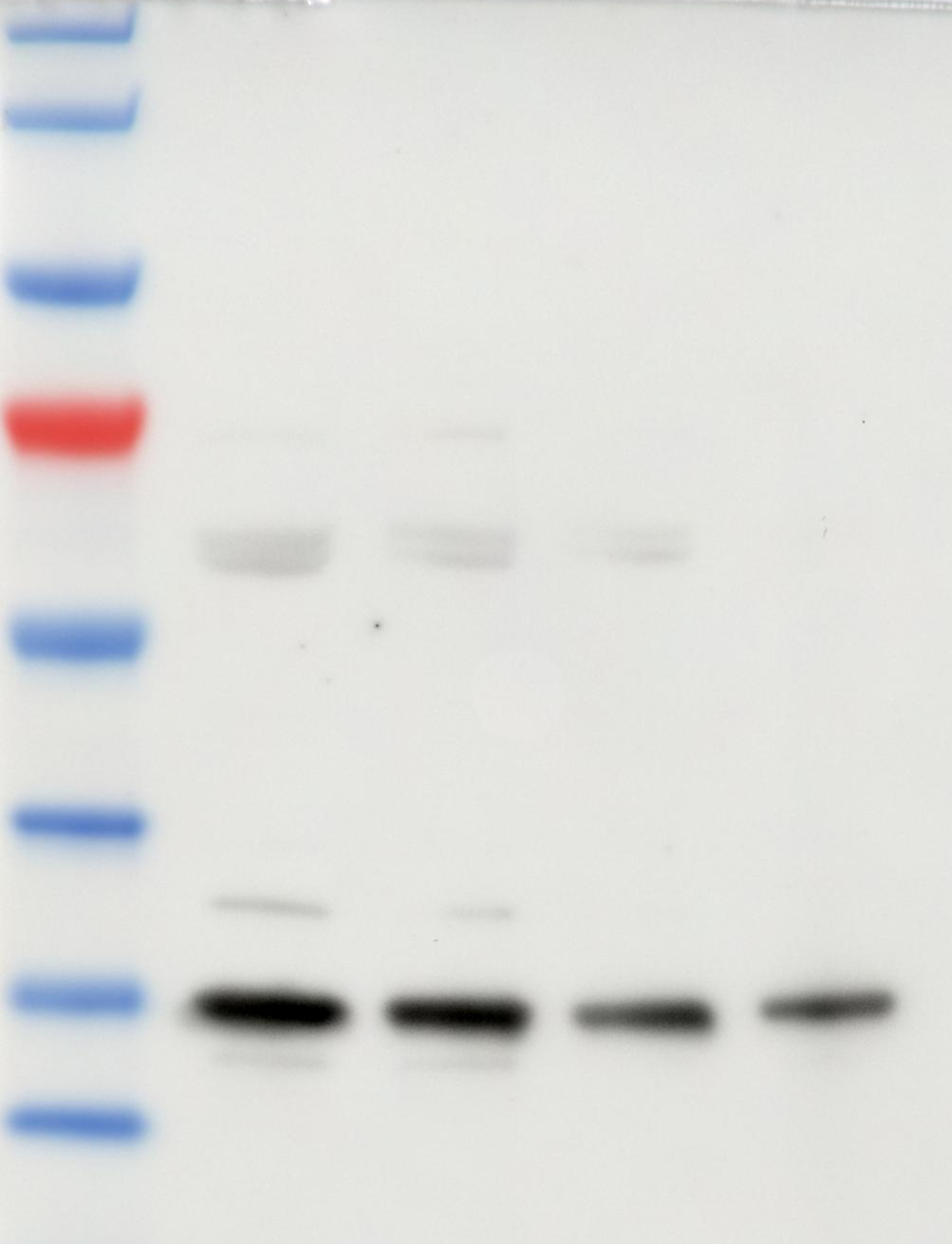

Supplement: Figure 8—source data 3. [file elife-101460-fig8-data3.zip › Fig. 8-source data3/Fig. 8k DCV.tif]

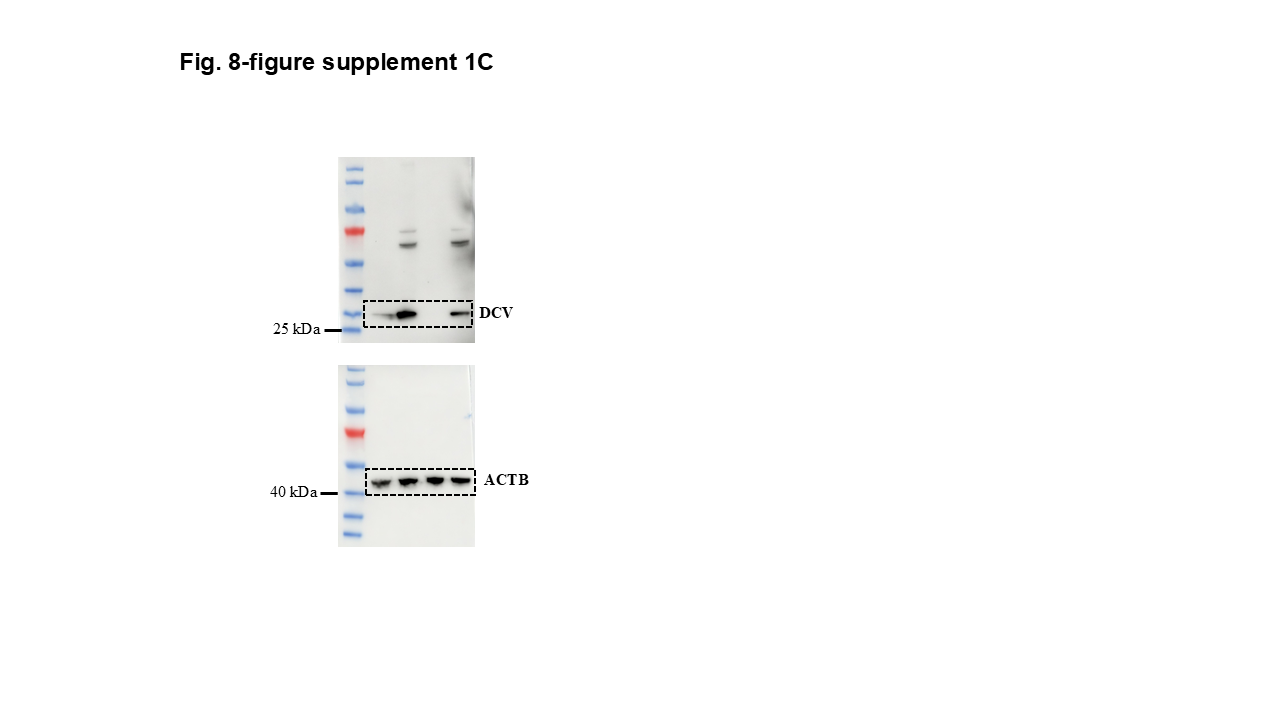

Supplement: Figure 8—figure supplement 1—source data 2. [file elife-101460-fig8-figsupp1-data2.zip › Fig. 8-figure supplement 1-source data2/Fig. 8-figure suppl 1-source data 2.tif]

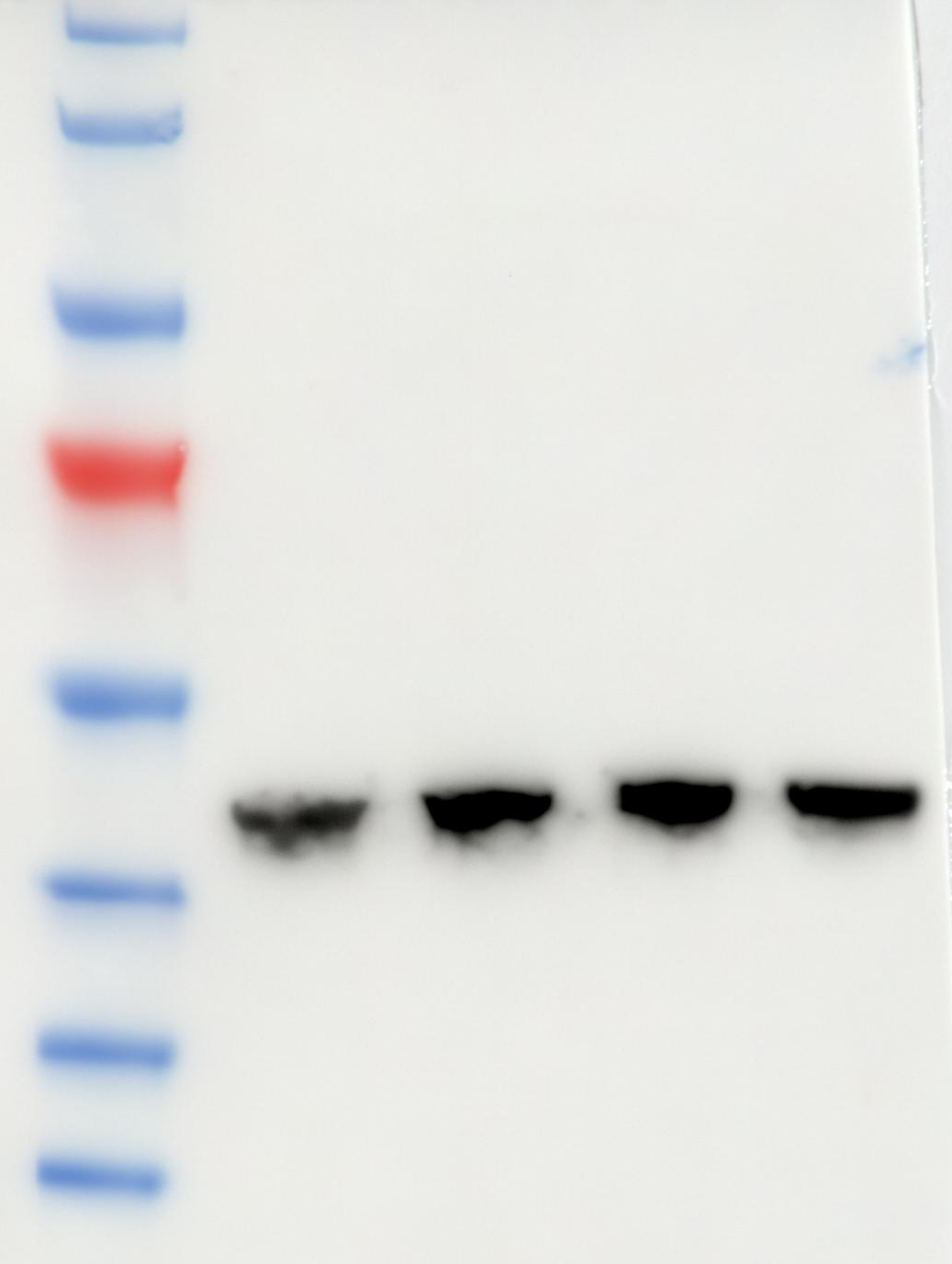

Supplement: Figure 8—figure supplement 1—source data 3. [file elife-101460-fig8-figsupp1-data3.zip › Fig. 8-figure supplement 1-source data3/Fig. 8-figure supplement 1C ACTB.tif]

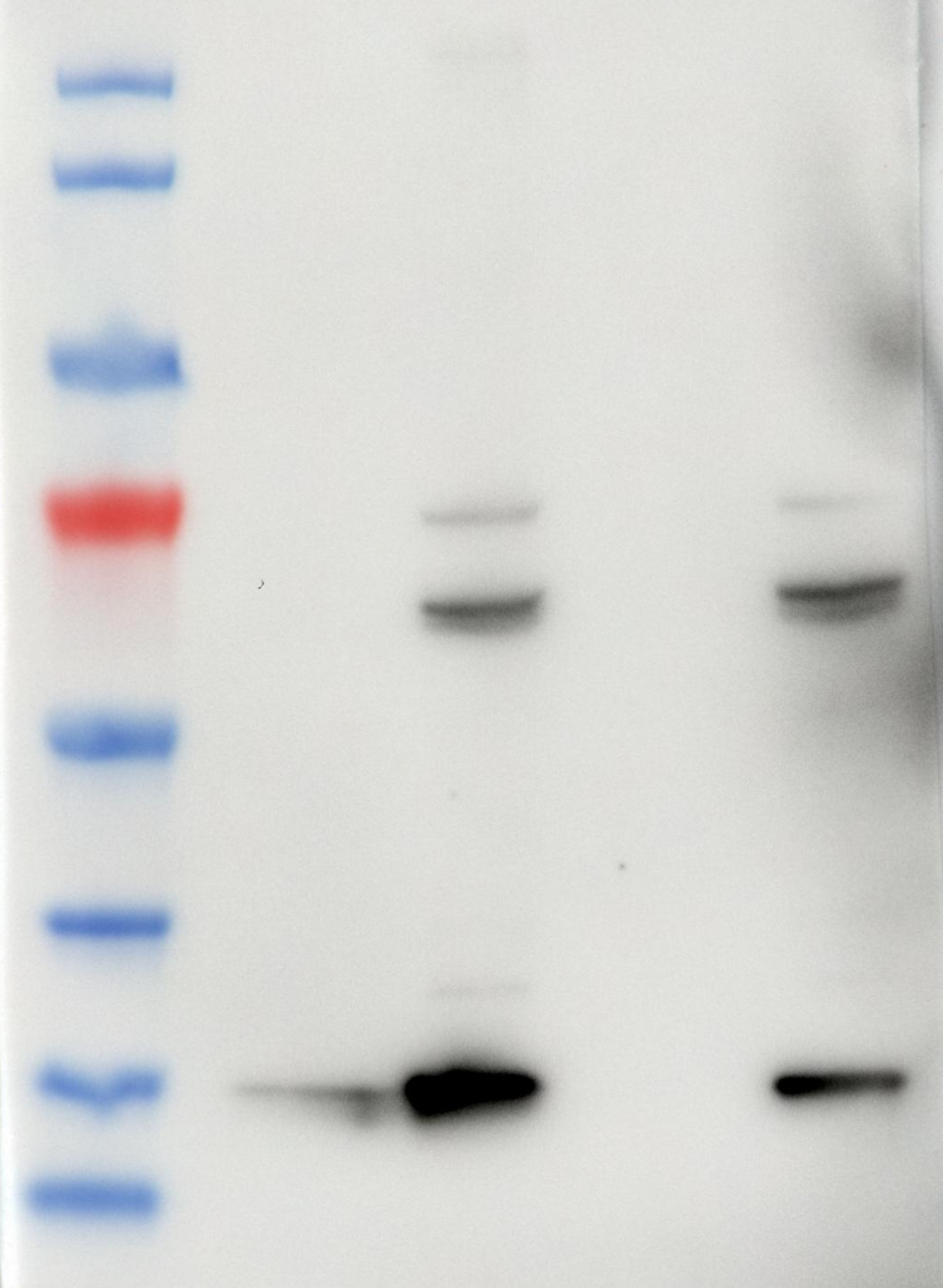

Supplement: Figure 8—figure supplement 1—source data 3. [file elife-101460-fig8-figsupp1-data3.zip › Fig. 8-figure supplement 1-source data3/Fig. 8-figure supplement 1C DCV.tif]
